# Impact of lockdown on the growth of children in China aged 3-6 years during the COVID-19 pandemic

**DOI:** 10.3389/fendo.2023.1301889

**Published:** 2024-01-03

**Authors:** Peiling Cai, Yuxuan Liu, Zhen Yang, Yueyao Luo, Yanqiong Zhang, Peng Ye, Xiaoling Yin, Nanying Xiao, Xinwei Chen, Mengping Wang, Beili Xiao, Hongying Zhao

**Affiliations:** ^1^ School of Preclinical Medicine, Chengdu University, Chengdu, Sichuan, China; ^2^ Clinical Medical College & Affiliated Hospital of Chengdu University, Chengdu, Sichuan, China; ^3^ Maternal and Child Health Service Center of Wuhua District, Kunming, Yunnan, China; ^4^ Department of Pediatrics, Zhongshan Hospital of Xiamen University, Xiamen University, Xiamen, Fujian, China

**Keywords:** lockdown, COVID-19 pandemic, height, weight, body mass index, preschool children

## Abstract

**Background:**

Lockdowns in COVID-19 pandemic led to less physical activity and more intake of unhealthy food in children. The aim of this study was to investigate the negative impact of major lockdowns on the growth of children aged 3-6 years during COVID-19 pandemic period.

**Methods:**

Physical examination results in 2019 to 2022 from 5834 eligible children (2972 males and 2862 females) from Southwestern China who were 3 years old in 2019 were retrospectively collected. Height and weight data points were extracted from the results, and percentiles of height (height%), weight (weight%), and BMI (BMI%), and rates of overweight and obesity were calculated and compared between different years during the pandemic.

**Results:**

After analyzing the 15404 growth data points from 5834 children, a slowly increasing trend of height% from 2019 to 2022 was observed. Weight%, BMI%, overweight rate, obesity rate, and combined overweight and obesity rate had two peaks in 2020 and 2022 when major lockdowns were adopted and a drop in between (year 2021), except for obesity rate which did not drop in 2021. Similar results were shown after stratification by gender.

**Conclusion:**

The lockdowns in COVID-19 pandemic promoted obesity of kindergarten children, but did not show any negative impact on their height growth possibly due to over-nutrition of children during lockdowns. More efforts need to be made to limit the increase of obesity rate in kindergarten children during possible future lockdowns.

## Introduction

The coronavirus disease 2019 (COVID-19) pandemic caused by the severe acute respiratory syndrome coronavirus 2 (SARS-CoV-2) started in late 2019. In January 2020, because of the wide spread of the disease worldwide, World Health Organization (WHO) declared a Public Health Emergency of International Concern over the COVID-19 outbreak (1). Due to the significant morbidity and mortality of the disease, most countries adopted different restriction measures to minimize its spreading, e.g., use of face masks, social distancing, COVID-19 screening and contact tracing, and quarantine measures including small-scale home quarantines, and more generalized lockdown of a district or a city.

During the lockdown period, residents were asked to stay at home. Like their parents who finished their work at home online, children also attended their classes online. This led to unhealthy behaviors, e.g., longer screen time, less physical activity, and more intake of unhealthy food in children (2–4), which could potentially cause negative impacts on their health and growth (5–7). Less physical activity and over-consumption of unhealthy food are risk factors of childhood obesity (8, 9), which may increase the risk of cardiovascular disease, metabolic disease, and cancer in their adulthood (10). A study from Poland reported a 2-fold increase of the risk of obesity or overweight in teenagers with less physical activities (11). A study from the Netherlands showed that sports participation was associated with less body fat among 6-year-old children (12). Study on the physical activities of children during COVID-19 lockdown also revealed less weight gain in physically-active children (13). Indeed, observational studies from many different countries (including the United States, Austria, Australia, Israel, Italy, Syria, the Netherlands, Jordan, and China) revealed increased rates of overweight and obesity in children during the COVID-19 pandemic and lockdown period (14–28). In addition, a previous study also found that height increase of kindergarten children was slower in 2020 compared to other years (27).

Current studies investigating the impact of COVID-19 lockdown mostly focused on adults or school-age children, and only two studies investigated the growth status of kindergarten children during COVID-19 lockdown (27, 28), leaving a significant knowledge gap in the potential impact of lockdowns on the growth of preschool children. Preschool or kindergarten period is a key developmental stage (29), and unhealthy behaviors during preschool period may lead to substantial impact on their health and development which could last for their whole life. For example, as indicated in a systemic review and meta-analysis, ~55% of obese children will continue to be obese in adolescence, and ~70% of obese adolescents will also be obese when they reach 30 (30). Results from another systemic review and meta-analysis showed that although with low predictive values, high childhood body mass index (BMI) was associated with higher incidence of adult diabetes, coronary heart disease, and cancer (31). It is therefore necessary to put more research effort on preschool children and investigate the impact of COVID-19 lockdown on this group of population.

During the COVID-19 pandemic, there were two major lockdowns adopted in China to limit the spreading of the disease. One was in 2020 soon after the pandemic started, and the other was in 2022 when the highly contagious COVID-19 variant, Omicron, was spreading across the world. Different from the lockdown in 2020 which was nationwide and lasted for more than 3 months, the lockdowns in 2022 were mostly sporadic (depending on where COVID-19-positive patients or carriers were identified), small in size, and short in time. But due to the high transmissibility of Omicron variant, there were a large number of small lockdowns in 2022. Two previous studies by Wen (27) and by Long (28) investigating the impact of lockdown on kindergarten children in China discussed the major lockdown in 2020 only, and impact of the large number of small lockdowns in 2022 in China was still unknown. Hypothesis of this study was that similar to the major lockdown in 2020, the lockdowns in 2022 which were small in scale but large in numbers could also have negative impacts on children’s health and growth by promoting weight gain and slowing the increase of height of kindergarten children. On the basis of previous studies, this study aimed to further analyze the growth status of kindergarten children from 2019 to 2022, in hopes of better understanding the potential impact of lockdowns on the growth of preschool children.

## Methods

### Collection of height and weight data and calculation of BMI and percentile

In order to better understand the growth of preschool children during the pandemic, a group of children who were 3 years old in 2019 were involved. From Maternal and Child Health Care Hospital of Wuhua District, Kunming City, Yunan Province, weight and height data of children were collected, who had their physical examination in any year between 2019 and 2022, and were 3 years old in 2019, 4 years old in 2020, 5 years old in 2021, or 6 years old in 2022. Height was measured using a stadiometer (HM1000-SZ, Jiangsu Hemei Electrical Technology, China). After removing hats and shoes, children were asked to stand on the scale with their two heels together, and their back, hip, and heels touching the rod. Height was then measured with accuracy of 0.1 centimeter. Weight was measured using a weight scale (M303, T-Scale, China). With light clothes and no shoes on, children were asked to stand steady on the middle of the scale. Weight was then measured with accuracy of 0.1 kilogram. Incomplete data (missing height or weight data) or data from children who were not 3 years old in 2019 (or not 4 years old in 2020, not 5 years old in 2021, or not 6 years old in 2022) were excluded. BMI was calculated by dividing the weight (in kilogram) by squared height (in meter) (kg/m^2^). Based on the latest growth standard for children under 7 years of age (32) issued by National Health Commission of People’s Republic of China, percentile of each height, weight, and BMI data points (height%, weight%, and BMI%) was calculated based on the reference population of the specific age (in month), using Microsoft Excel. Overweight and obesity rates were also calculated based on the definition of overweight (BMI% ≥ +1 standard deviation and < +2 standard deviation) and obesity (BMI% ≥ +2 standard deviation) in the latest growth standard for children under 7 years of age (32). This study was approved by Institutional Review Board of Affiliated Hospital of Chengdu University (approval No.: PJ2023-019-03). Informed consent was waived by the Institutional Review Board since this study was retrospective and non-interventional. All data were collected and analyzed anonymously.

### Statistical analysis

Since data were not normally distributed, comparison of height%, weight%, and BMI% between different years were performed using Kruskal-Wallis test and *post hoc* Mann-Whitney test. In children with paired data, the height%, weight%, and BMI% were also compared between adjacent years (2019 *versus* 2020, 2020 *versus* 2021, and 2021 *versus* 2022) using Wilcoxon Signed Rank test. Trend test was performed using Jonckheere Terpstra test. Comparison of overweight and obesity rates between different years were performed using Chi-square test. All the statistical analysis was performed using IBM SPSS 19.0 (IBM). *P* < 0.05 was considered statistically significant.

## Results

### Demographic characteristics

In all, 15404 growth data points were collected (5523 in year 2019, 4275 in year 2020, 4457 in year 2021, and 1149 in year 2022) from 5834 eligible children (2972 males and 2862 females). The dropped number of physical examination data points in the pandemic period could possibly be due to the reduced service time and capacity of physical examination center (e.g., suspension of physical examination service during major lockdowns, and decreased service capacity to ensure social distancing) which stopped some children from attending regular physical examination.

### Changes of height%, weight%, and BMI% from 2019 to 2022

**Figure 1:**
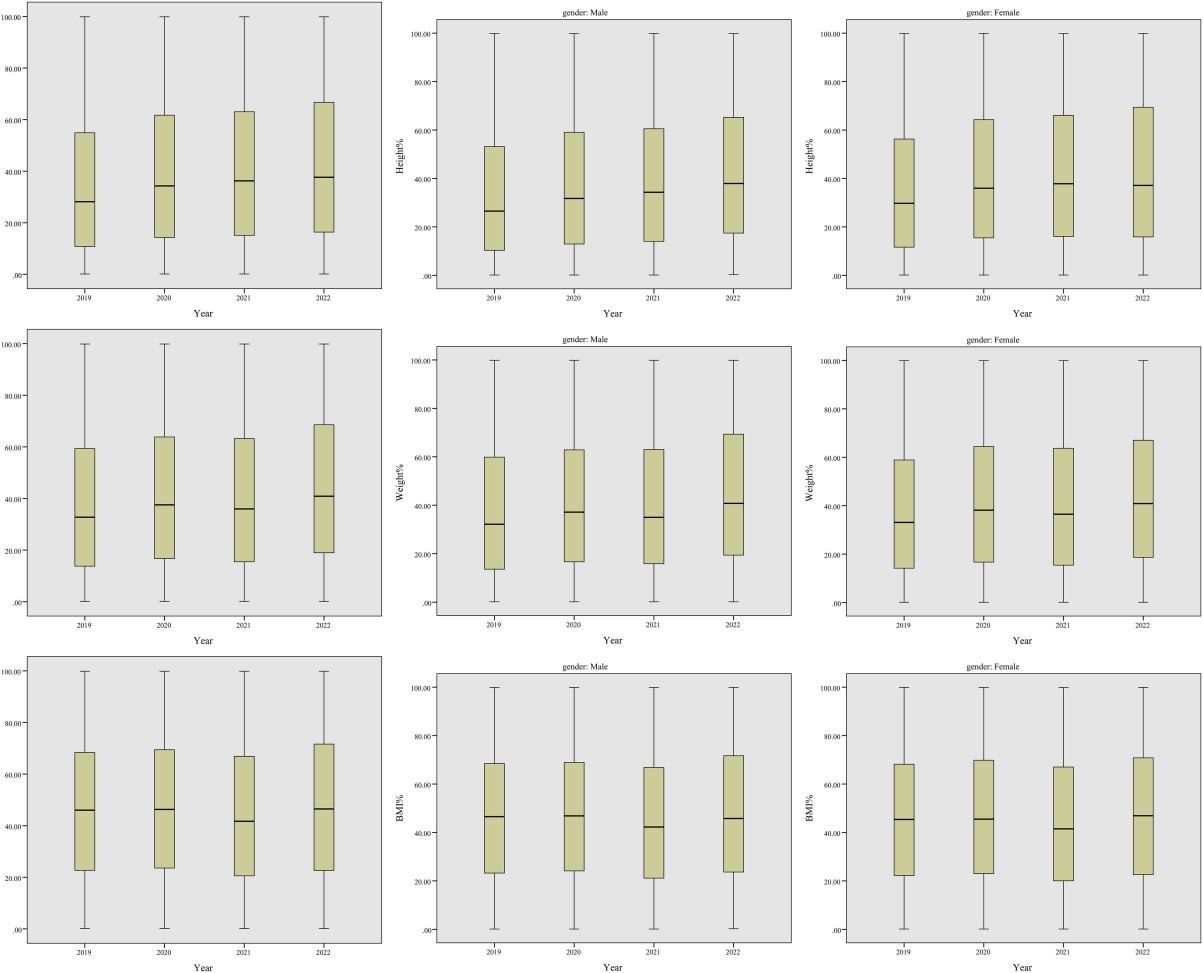
Box plots of height%, weight% and BMI% from 2019 to 2022, before and after stratification by gender.

As shown in **Figure 1** and **Table 1**, from all the data collected, the median height% showed an overall increasing trend (*P* < 0.001), from 28.16% in 2019 to 37.65% in 2022. Starting from 32.75% in 2019, median weight% showed two peaks in 2020 (37.52%, *P* < 0.001 compared to 2019) and 2022 (40.87%, *P* < 0.001 compared to 2021) when the two major lockdowns were adopted, with a lower median percentile in between (35.94% in 2021, *P* = 0.162 compared to 2020). Median BMI% was quite stable in the 4 years except for a drop in 2021 (41.71%, *P* < 0.001 compared to 2020) which could be due to the drop of weight% in that year. After stratification by gender, these changes of height%, weight%, and BMI% between adjacent years were similar. Overall, male children showed lower height% and weight% in all the four years compared to female children. BMI% of male children was higher than female children in the four years except 2022.

**Table 1:** Height%, weight% and BMI% from 2019 to 2022.

	Median (inter-quartile range) (%)				*P* value (Kruskal-Wallis test)	*P* value (*post hoc* analysis)		
	2019	2020	2021	2022		2019 *versus* 2020	2020 *versus* 2021	2021 *versus* 2022
Overall								
Height%	28.16 (44.07)	34.28 (47.46)	36.26 (47.93)	37.65 (50.44)	< 0.001	< 0.001	0.023	0.074
Weight%	32.75 (45.58)	37.52 (47.19)	35.94 (47.74)	40.87 (49.66)	< 0.001	< 0.001	0.162	< 0.001
BMI%	46.01 (45.73)	46.32 (45.92)	41.71 (46.33)	46.50 (49.01)	< 0.001	0.182	< 0.001	0.001
Male								
Height%	26.52 (42.85)	31.74 (46.19)	34.28 (46.51)	37.91 (47.85)	< 0.001	< 0.001	0.036	0.024
Weight%	32.14 (46.25)	37.17 (46.23)	35.03 (47.19)	40.76 (50.04)	< 0.001	< 0.001	0.436	0.001
BMI%	46.53 (45.24)	46.82 (44.81)	42.24 (45.74)	45.78 (48.19)	0.002	0.455	< 0.001	0.020
Female								
Height%	29.73 (44.61)	35.98 (48.82)	37.81 (50.03)	37.13 (53.65)	< 0.001	< 0.001	0.241	0.781
Weight%	33.09 (44.67)	38.14 (47.73)	36.49 (48.33)	40.87 (48.80)	< 0.001	< 0.001	0.236	0.014
BMI%	45.36 (46.08)	45.51 (46.87)	41.48 (47.00)	46.92 (48.33)	0.007	0.269	0.003	0.011

### Change of height%, weight%, and BMI% between adjacent years in children with paired data points

After the analysis on all the collected data points, changes of height%, weight%, and BMI% between two adjacent years in children with data points in both years (paired data points) were analyzed. As shown in **Table 2**, height% kept increasing from 2019 to 2022, but the increasing amount was getting smaller. Weight% also showed significant increases from 2019 to 2020 (1.89%, *P* < 0.001), and from 2021 to 2022 (2.54%, *P* < 0.001), with a significant drop in between (-0.87% from 2020 to 2021, *P* < 0.001). Possibly due to the drop in weight% in 2021, BMI% also showed a significant drop (-2.33%) from 2020 to 2021 (*P* < 0.001), and then rose by 3.31% from 2021 to 2022 (*P* < 0.001). Similar changes between adjacent years were observed in male and female children after stratification, except that height% of female children slightly dropped from 2021 to 2022 (-0.13%, *P* = 0.179).

**Table 2:** Height%, weight% and BMI% of children with paired data points in adjacent years.

	From 2019 to 2020		From 2020 to 2021		From 2021 to 2022	
	Median change (inter-quartile range, 95% confidence interval) (%)	*P* value	Median change (inter-quartile range, 95% confidence interval) (%)	*P* value	Median change (inter-quartile range, 95% confidence interval) (%)	*P* value
Overall						
N	4266		3766		1076	
Height%	2.71 (9.45, 2.45 ~ 2.98)	< 0.001	0.76 (7.12, 0.59 ~ 0.92)	< 0.001	0.07 (6.42, -0.16 ~ 0.33)	0.741
Weight%	1.89 (13.38, 1.52 ~ 2.22)	< 0.001	-0.87 (10.24, -0.11 ~ -0.59)	< 0.001	2.54 (10.18, 2.02 ~ 3.01)	< 0.001
BMI%	0.35 (20.79, 0.00 ~ 0.71)	0.018	-2.33 (15.59, -2.82 ~ -1.85)	< 0.001	3.31 (16.05, 2.46 ~ 4.06)	< 0.001
Male						
N	2177		1933		547	
Height%	2.26 (9.06, 1.91 ~ 2.63)	< 0.001	0.81 (6.79, 0.59 ~ 1.02)	< 0.001	0.29 (6.15, -0.19 ~ 0.66)	0.375
Weight%	1.56 (12.92, 1.22 ~ 1.96)	< 0.001	-1.15 (10.16, -1.60 ~ -0.80)	< 0.001	2.55 (9.66, 2.07 ~ 3.43)	< 0.001
BMI%	0.34 (20.75, -0.18 ~ 0.78)	0.089	-2.54 (15.64, -3.29 ~ -1.97)	< 0.001	2.99 (15.69, 1.65 ~ 4.07)	< 0.001
Female						
N	2089		1833		529	
Height%	3.19 (9.89, 2.81 ~ 3.62)	< 0.001	0.69 (7.56, 0.42 ~ 0.94)	< 0.001	-0.13 (6.55, -0.73 ~ 0.18)	0.179
Weight%	2.32 (13.63, 1.85 ~ 2.73)	< 0.001	-0.53 (10.17, -0.93 ~ -0.27)	< 0.001	2.44 (10.57, 1.59 ~ 3.39)	< 0.001
BMI%	0.35 (20.78, -0.16 ~ 0.94)	0.096	-2.04 (15.92, -2.74 ~ -1.59)	< 0.001	3.52 (16.19, 2.22 ~ 4.63)	< 0.001

### Changes of overweight and obesity rates from 2019 to 2022

After the analysis of height%, weight%, and BMI%, overweight and obesity rates of the children were also investigated. As shown in **Table 3**, the combined rate of overweight and obesity significantly increased from 2019 to 2020 (1.84%, *P* = 0.005) and from 2021 to 2022 (2.84%, *P* = 0.010), with a slight drop in between (-0.85%, from 2020 to 2021, *P* = 0.287). The overweight rate showed a similar drop from 2020 to 2021 (-0.77%, *P* = 0.224) and a significant increase from 2021 to 2022 (2.04%, *P* = 0.036), but only showed a slight increase from 2019 to 2020 (0.17%, *P* = 0.784). Obesity rate showed a significant increase from 2019 to 2020 (1.68%, *P* < 0.001), and was stable from 2020 to 2021 (0.01%, *P* = 0.975), followed by a slight increase from 2021 to 2022 (0.80%, *P* = 0.169).

**Table 3:** Overweight and obesity rates from 2019 to 2022.

	Rate (%)				*P* value (Chi-square test)			
	2019	2020	2021	2022	2019 *versus* 2020	2020 *versus* 2021	2021 *versus* 2022	
Overall								
Overweight	9.87	10.04	9.27	11.31	0.784	0.224	0.036	
Obesity	1.34	3.02	3.03	3.83	< 0.001	0.975	0.169	
Overweight + obesity	11.21	13.05	12.30	15.14	0.005	0.287	0.010	
Male								
Overweight	9.62	9.98	9.11	10.65	0.671	0.319	0.254	
Obesity	1.68	3.39	3.05	4.47	< 0.001	0.521	0.089	
Overweight + obesity	11.30	13.37	12.16	15.12	0.027	0.224	0.056	
Female								
Overweight	10.12	10.09	9.44	11.99	0.973	0.472	0.070	
Obesity	0.99	2.63	3.01	3.17	< 0.001	0.459	0.836	
Overweight + obesity	11.11	12.72	12.44	15.17	0.086	0.784	0.086	

After stratification by gender, combined overweight and obesity rate also showed two peaks in 2020 and 2022 in both male and female children. Similarly, in both genders, overweight rate dropped from 2020 to 2021 (male: -0.87%, *P* = 0.319; female: -0.65%, *P* = 0.472) and increased from 2021 to 2022 (male: 1.54%, *P* = 0.254; female: 2.55%, *P* = 0.070). Obesity rate also showed significant increases from 2019 to 2020 in both male (1.71%, *P* < 0.001) and female children (1.64%, *P* < 0.001). From 2020 to 2021, obesity rate of female children slightly increased (0.38%, *P* = 0.459), while obesity rate of male children slightly decreased (-0.34%, *P* = 0.521).

## Discussion

Although lockdown helped to minimize the spreading of the virus during outbreaks in COVID-19 pandemic, it could also bring negative impacts to the residents, including kindergarten children. On the basis of previous studies, this study investigated the impact of the two major lockdowns in 2020 and 2022 on the growth of children aged 3-6 years.

From the analysis of all the collected data, a continuous but more and more slowly increasing trend of height% from 2019 to 2022 was observed, which could be caused by increased nutrition intake during the lockdowns. For example, an international online survey reported unhealthier food consumption and meal patterns during lockdown period, including consumption of unhealthy food, eating out of control, more snacks between meals, and increasing number of meals (2). This observation is different from the observation of Wen’s study that average increase in height was about 1 centimeter less as compared with the same period in the past two years (27). Since Wen’s study used a different study design by comparing children of the same age in several continuous years (e.g. 3-year old children in 2018, 2019, 2020, and 2021), it is difficult to directly compare their results with the results of this study. Instead of using unpaired data points as in Wen’s study (27), this study tracked the growth of children who were 3 years old in 2019, and most of the data points were paired between years (except for the missing data), which could help minimize the bias caused by different participants between groups. From the analysis results on paired data points, an increasing trend of height% from 2019 to 2022 was also observed, further convincing the findings in all the collected data. Furthermore, the discrepancy between Wen’s study and this study might also be caused by different reference growth standards used. Instead of using the growth standards from WHO as in Wen’s study (27), the newly released growth standards of children under 7 years of age (32) from China government was used in this study as the reference of calculation, which should be more suitable for an investigation on a Chinese population.

Regarding the weight and BMI of children, significantly higher weight% and BMI% in 2020 and 2022 were found in paired data points. In all the data collected, peaked weight% and BMI% were also observed in 2020 and 2022, although the differences were not always significant between adjacent years. Similar peaks in 2020 and 2022 were also observed in combined rate of overweight and obesity. Interestingly, when analyzed separately, obesity rate increased significantly from 2019 to 2020 but was stable from 2020 to 2021, when overweight rate only showed a slight increase from 2019 to 2020 and then dropped from 2020 to 2021, indicating that lockdowns had more negative impact on obesity rate than overweight rate. Clinicians should pay more attention on the obese children since it seems to be difficult for them to return to overweight or normal weight after a lockdown period. Compared to the previous studies by Wen (27) and by Long (28), this study showed lower overweight rate (overall: 9.87% ~ 11.31% *versus* 14.07% ~ 17.43% (27); boys: 9.11% ~ 10.65% *versus* 14.3% ~ 18.2% (28); girls: 9.44% ~ 11.99% *versus* 10.6% ~ 13.9% (28)) and obesity rates [overall: 1.34% ~ 3.83% *versus* 10.47% ~ 12.28% (27); boys: 1.68% ~ 4.47% *versus* 6.6% ~ 9.5% (28); girls: 0.99% ~ 3.17% *versus* 2.8% ~ 4.5% (28)]. This could be due to the different population involved in these three studies. Population investigated in this study was from southwestern part of China, while population of Long’s study was from eastern part of China (28) and population of Wen’s study was from southwestern, eastern, and southern parts of China (27). Although showing different overweight and obesity rates, all of these three studies showed increased obesity rates during lockdown periods (from 2019 to 2020: from 9.87% to 10.04% in this study, from 10.47% to 12.28% in Wen’s study (27), and from 6.6% to 9.5% in boys and from 2.8% to 4.5% in girls in Long’s study (28); from 2021 to 2022: from 3.03% to 3.83% in this study), further demonstrating that lockdown could promote obesity of kindergarten children, possibly through unhealthy eating behaviors (e.g. over-nutrition, consumption of unhealthy food, and increased numbers of snacks or meals) (2), as well as less physical activity which is a risk factor for childhood obesity (8–13). In addition, results of this study also indicated that although quite different from the lockdown in 2020 in size, time, and number, the lockdowns in 2022 also had similar negative impacts on children’s growth.

Furthermore, the data were also stratified by gender and changes of growth parameters in male and female children were also investigated. Changes in height%, weight%, BMI%, and overweight and obesity rates were similar after stratification, in both male and female children. In both genders, height% continuously increased from 2019 to 2022, except for a slightly decrease observed from 2021 to 2022 in female children. Two peaks were also observed in weight% and BMI% in 2020 and 2022 in both genders, with a drop in between, although the differences were not always significant. Combined overweight and obesity rate also peaked in 2020 and 2022 in both genders, and there were also significant increases in obesity rates from 2019 to 2020 in both male and female children, which was similar as the increase of obesity rate from 2019 to 2020 in Long’s study (boys: 6.6% to 9.5%; girls: 2.8% to 4.5%) (28). Increases in overweight rate from 2021 to 2022 were also observed in both male and female children (male children: 9.11% to 10.65%; female children: 9.44% to 11.99%), which was not reported in previous studies. Different from overall results, there was a slight increase (2.63% to 3.01%) in obesity rate in female children from 2020 to 2021, accompanied by a slight decrease in male children (3.39% to 3.05%).

## Conclusions

In summary, using paired data points from a group of children aged 3 years in 2019, this study investigated the impact of major lockdowns on the growth of children aged 3-6 years, which covered the whole COVID-19 pandemic period from 2019 to 2022. This study was the first to use paired data points to investigate the impact of lockdowns on kindergarten children, which helped to minimize the bias caused by different participants between groups. In addition, this study was also the first to investigate the influence of large number of small lockdowns in China in 2022 on the growth of kindergarten children. Results of this study indicated that major lockdowns in COVID-19 pandemic did not delay but slightly promoted the height growth of kindergarten children, possibly due to over-nutrition of children during lockdowns. Similar to the previous studies, the weight and BMI percentiles and combined rates of overweight and obesity showed peaks during the major lockdown periods (including the large lockdown in 2020 and large number of small lockdowns in 2022), further demonstrating that major lockdowns may promote obesity in kindergarten children. In addition, after the significant increase during the major lockdown in 2020, the obesity rate of the children did not drop in 2021, indicating that major lockdowns may have long-term impacts on the obesity rate of children. Therefore, during possible major lockdowns in the future, more efforts are required to increase the physical activities of kindergarten children, limit their consumption of unhealthy food, and control their number of meals, especially in overweight and obese children. Limitations of this study may be that the growth data were collected from a single center and therefore may not represent the whole population of kindergarten children in China. In addition, weight and height data were retrospectively collected and measurement was performed by different operators, which may lead to potential bias. Studies with multiple centers from the whole country will be needed to further demonstrate the impact of major lockdowns on kindergarten children.

## Data availability statement

The raw data supporting the conclusions of this article will be made available by the authors, without undue reservation.

## Ethics statement

The studies involving humans were approved by Ethics committee, Affiliated Hospital of Chengdu University. The studies were conducted in accordance with the local legislation and institutional requirements. The ethics committee/institutional review board waived the requirement of written informed consent for participation from the participants or the participants’ legal guardians/next of kin because this research is a retrospective study that will not have adverse effects on the health of the participants. Furthermore, the privacy and personal identity information of the participants are protected by removing personal information characteristics.

## Author contributions

PC: Conceptualization, Data curation, Funding acquisition, Writing – original draft. YXL: Data curation, Writing – original draft, Investigation, Resources. ZY: Data curation, Writing – original draft. YYL: Data curation, Writing – original draft. YZ: Writing – original draft, Resources. PY: Methodology, Writing – original draft. XY: Writing – original draft, Resources. NX: Writing – original draft, Resources. XC: Writing – original draft, Software. MW: Data curation, Writing – original draft. BX: Data curation, Writing – original draft. HZ: Writing – review & editing.
